# Editorial: Molecular pathogenesis of tropical veterinary diseases

**DOI:** 10.3389/fvets.2023.1163154

**Published:** 2023-04-04

**Authors:** Sarah Othman, Zunita Zakaria, Mohd Effendy Abd Wahid

**Affiliations:** ^1^Department of Cell and Molecular Biology, Faculty of Biotechnology and Biomolecular Sciences, Universiti Putra Malaysia, Serdang, Malaysia; ^2^Universiti Putra Malaysia - Majlis Kanser Negara / National Cancer Council Malaysia Cancer Research Laboratory, Institute of Bioscience, Universiti Putra Malaysia, Serdang, Malaysia; ^3^Department of Veterinary Pathology and Microbiology, Faculty of Veterinary Medicine, Universiti Putra Malaysia, Serdang, Malaysia; ^4^Institute of Marine Biotechnology, Universiti Malaysia Terengganu, Kuala Terengganu, Malaysia

**Keywords:** tropical veterinary diseases, molecular pathogenesis, control, prevention, vaccine

Tropical veterinary diseases remain a major global concern for the health and well-being of animals and humans. The hot-humid climate in many tropical and subtropical regions promotes the spread of diseases caused by pathogens such as viruses, bacteria, parasites, and fungi. Some of the tropical veterinary diseases include foot-and-mouth disease (FMD), hemorrhagic septicemia (HS), Newcastle disease, influenza, rabies, malaria, and dengue fever. The prevalence of tropical veterinary diseases is high in many parts of these regions, particularly in rural communities that rely on livestock for food and income.

These diseases also pose risk to human health, either through direct contact with infected animals or through indirect contact with contaminated fomites, food, or water. Circumstantial factors, such as poverty, restricted access to veterinary facilities, and lack of preventative measures, have worsened the situation. The impact of these diseases can be significant, leading to economic losses for the livestock industry, and imposing severe threats to existing and future food security. To address this issue, effective control and prevention strategies, such as awareness of tropical veterinary diseases among the various stakeholders, improved biosecurity measures, strengthened veterinary services in affected areas, improved innovative technologies by incorporating surveillance AI in diagnostic tools, and sustainable vaccine programs, must be implemented. Nevertheless, there is still much work to be done to effectively address the situation, and ongoing research and strategic collaboration from affected countries are crucially needed.

The advancement of modern biotechnology, including molecular biology techniques, has significantly contributed to the understanding of the pathogenesis of infectious diseases, specifically tropical veterinary diseases. Molecular pathogenesis research includes the study of the molecular mechanisms underlying the development and progression of a disease, and it has become a critical tool in the control and prevention of diseases. With this, it is possible to study host–pathogen interactions and host immune responses, develop new vaccines and treatments, and improve monitoring and surveillance. For instance, molecular pathogenesis research has led to the discovery of new vaccine targets, the development of novel vaccine platforms, and the identification of new drug targets for the treatment of these diseases.

In captive bird populations, such as in zoos, the presence of mosquitoes and other vectors can facilitate the transmission of avian malaria, leading to outbreaks of the disease. In this collection, Iurescia et al. conducted a multidisciplinary study to understand the cause of death of captive African penguins (*Spheniscus demersus*) infected by avian malaria. The approach includes pathological examinations of the penguins' tissues, molecular characterization of the *Plasmodium* species and lineages, and mosquito surveillance. The investigation reveals that *P. matutinum* was responsible for the deaths, and *Culex pipiens* mosquito, which is present in the region, was involved in the transmission of the disease. The study provides important information about the cause of death and highlights the importance of monitoring and preventing the spread of avian malaria in captive populations of African penguins.

Moreover, Wardhani et al. investigated the presence of viral neuropathogens in the brains of rabies-negative cats and dogs that were submitted to the Thai Red Cross Society (TRCS) with neurological signs before death. Based on the selective viral molecular screening, the study found that various viral neuropathogens were detected, including carnivore protoparvovirus-1 (CPPV-1) [canine parvovirus (CPV) and feline parvovirus (FPV)], feline calicivirus (FCV), feline bocavirus (FBoV), feline alphaherpesvirus-1 (FeHV-1), and canine distemper virus (CDV). Immunohistochemistry was also employed to further investigate the localization of each viral pathogen. The results revealed that CPPV-1 was present in most of the brain samples, which may have contributed to the neurological signs observed in the animals. The study highlights the importance of considering other viral pathogens in addition to rabies in the diagnosis of neurological diseases in animals.

Huang et al. elucidated the role of a specific amino acid in the non-structural protein 1 (NS1) on the virulence of the H5N6 influenza A virus (IAV). The study analyzed the pathogenicity of the H5N6 IAV strains isolate A/duck/Hubei/WH18/2015 (JX) and A/chicken/Hubei/XG18/2015 (XGD) in mice. Accordingly, the JX strain was more virulent than the XGD strain with substantial weight loss, severe damage to the lungs and heart, and increased lethality being observed in infected mice. Moreover, the pathogenicity of recombinant viruses demonstrated that 139D mutation in the NS1 could potentially have an impact on the virulence of the JX strain in mice. This study highlights that the amino acid 139D is significant for the virulence of the virus in mice and could serve as a marker for identifying different variants of the influenza virus.

In recent years, the expression of circular RNAs (circRNAs) has been extensively studied especially for its biological functions in human cells. In the study reported by Liu et al., differentially expressed circular RNAs (circRNAs) were identified in various liver cells, including hepatic stellate cells (HSCs), hepatocytes (HCs), and Kupffer cells (KCs), after infection with the *Echinococcus multilocularis* parasite. The findings were validated using quantitative real-time polymerase chain reaction (qRT-PCR) on a selected set of circRNAs. The study also aimed to explore the circRNA–microRNA–mRNA network in these cells during the infection. The result found that the expression of certain circRNAs in the liver cells was changed after infection and that some of the circRNAs had a role in regulating the expression of microRNAs and mRNAs, which in turn play a role in the host response to the parasite. The study highlights the importance of understanding the role of circRNAs in the host response to parasitic infections, which may provide insights into the development of new therapeutic strategies.

The giant river prawn, *Macrobrachium rosenbergii*, is highly exported and one of the most economically important seafood in Asia. Zhuo et al. analyzed the complete genome of highly pathogenic non-O1/O139 *Vibrio cholerae* strain GXFL1-4, isolated from freshwater prawn, *Macrobrachium rosenbergii*. The study identified the genes that contribute to the pathogenicity and antibiotic resistance of this strain. Based on the prediction, the genome contained multiple genes associated with pathogenicities, such as those involved in motility, adhesion, and toxin production. Multiple antibiotic-resistant genes, including kanamycin, ampicillin, tetracycline, aminoglycosides, and beta-lactams, were identified. The study provides valuable insights into the genetics of this strain of *V. cholerae* and highlights the presence of genes associated with pathogenicity and antibiotic resistance. The findings may aid in the development of more effective control and mitigation strategies for the disease.

In terms of surveillance in tropical veterinary diseases, Wu et al. investigated integrated vector surveillance in Zhejiang Province, China, in 2020, to monitor the occurrence of tropical vector-borne diseases (VBDs). The aim was to identify potential vectors of VBDs, as well as investigate the pathogen infection rates among arthropod vectors and reservoir hosts, including mosquitoes, rodents, ticks, and chigger mites. A pathogen infection survey on mosquitoes and rodents and a drug resistance survey on *Aedes albopictus* were conducted. The survey demonstrated that the primary vectors of tropical VBDs in the region were mosquitoes and ticks and that the pathogens and reservoir hosts were present in certain areas of the province. The study highlights the importance of integrated vector surveillance programs in the early detection and control of tropical vector-borne diseases.

Thus, the study of molecular pathogenesis plays a crucial role in enhancing the understanding of tropical veterinary diseases and developing new strategies for control and prevention. Studying the molecular basis of diseases helps in developing more accurate diagnostic methods, effective treatments, and strategies for disease prevention, leading to improved health and wellbeing of animals and humans in tropical regions. This collection will be beneficial in adding an in-depth insight into the molecular pathogenesis of tropical veterinary diseases.

## Author contributions

All authors listed have made a substantial, direct, and intellectual contribution to the work and approved it for publication.

